# Snapshots of human anatomy, locomotion, and behavior from Late Pleistocene footprints at Engare Sero, Tanzania

**DOI:** 10.1038/s41598-020-64095-0

**Published:** 2020-05-14

**Authors:** Kevin G. Hatala, William E. H. Harcourt-Smith, Adam D. Gordon, Brian W. Zimmer, Brian G. Richmond, Briana L. Pobiner, David J. Green, Adam Metallo, Vince Rossi, Cynthia M. Liutkus-Pierce

**Affiliations:** 1 Department of Biology, Chatham University, Pittsburgh, PA 15232 USA; 2 Center for the Advanced Study of Human Paleobiology, The George Washington University, Washington, DC, 20037 USA; 3 Department of Anthropology, Lehman College, New York, NY 10468 USA; 4 Department of Anthropology, CUNY Graduate Center, New York, NY 10016 USA; 5 Division of Paleontology, American Museum of Natural History, New York, NY 10024 USA; 6 Department of Anthropology, University at Albany, Albany, NY 12222 USA; 7 Department of Geological and Environmental Sciences, Appalachian State University, Boone, NC 28608 USA; 8 San Francisco, CA, 94102 USA; 9 Human Origins Program, Smithsonian Institution, Washington, DC, 20560 USA; 10 Department of Anatomy, Campbell University School of Osteopathic Medicine, Buies Creek, NC 27506 USA; 11 Digitization Program Office, Smithsonian Institution, Washington, DC, 20013 USA

**Keywords:** Biological anthropology, Palaeontology

## Abstract

Fossil hominin footprints preserve data on a remarkably short time scale compared to most other fossil evidence, offering snapshots of organisms in their immediate ecological and behavioral contexts. Here, we report on our excavations and analyses of more than 400 Late Pleistocene human footprints from Engare Sero, Tanzania. The site represents the largest assemblage of footprints currently known from the human fossil record in Africa. Speed estimates show that the trackways reflect both walking and running behaviors. Estimates of group composition suggest that these footprints were made by a mixed-sex and mixed-age group, but one that consisted of mostly adult females. One group of similarly-oriented trackways was attributed to 14 adult females who walked together at the same pace, with only two adult males and one juvenile accompanying them. In the context of modern ethnographic data, we suggest that these trackways may capture a unique snapshot of cooperative and sexually divided foraging behavior in Late Pleistocene humans.

## Introduction

Footprints are often ephemeral but when preserved in the geological record, these ichnofossils can provide unique snapshots of the lives of ancient organisms. Fossil tracks are generated and preserved on far shorter time scales than other common forms of fossil data (e.g., skeletal fossils), leading to a distinct set of hypotheses that can be developed and tested with this form of evidence. In paleoanthropology, researchers have analyzed fossil hominin footprints through a variety of analytical approaches that address a wide range of research questions (many are reviewed by Bennett and Morse^1^). Perhaps most common are analyses that derive inferences regarding hominin body size and size variation^2–4^, or foot anatomy, foot function and/or locomotion^4–17^. However, since footprint assemblages typically form on short time scales, these data can also be used to infer group composition and other behaviors of individuals who must have lived on the same landscape at the same time^16,18–23^.

Here, we report on Late Pleistocene human footprints discovered at Engare Sero, Tanzania. While our previous publications focused on the geological context and preservation of this site^24,25^, we explore the paleoanthropological implications of this remarkable assemblage of more than 400 human footprints. This includes inferences regarding the body sizes, locomotor behaviors, and composition of the group of humans who generated these tracks.

### Site description

**Figure 1:**
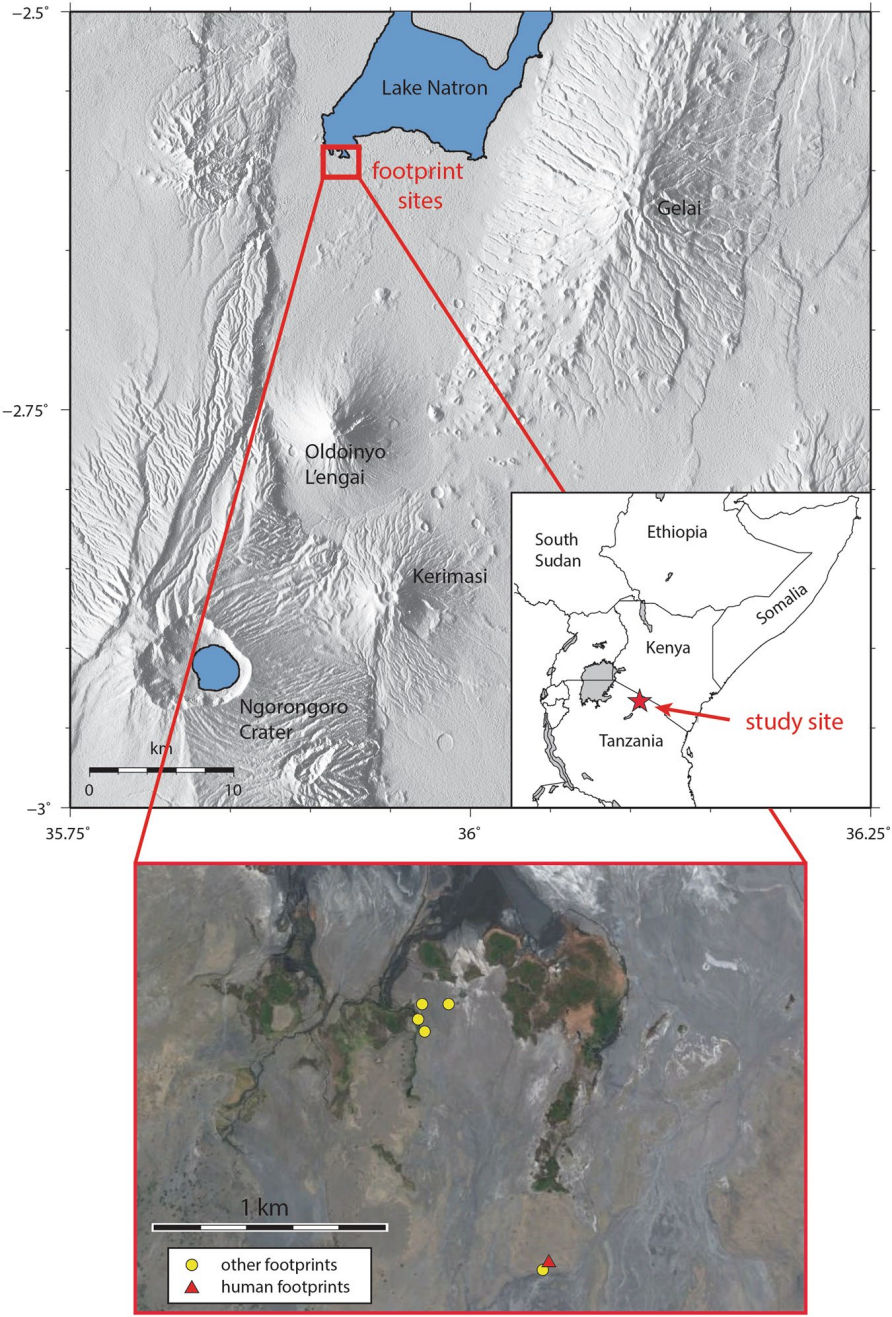
Map of the Lake Natron region, showing the location of the Engare Sero Footprint site, noting specific locations of both human and other animal footprint assemblages. Figure is adapted from^24^.

The Engare Sero footprint site lies just south of Lake Natron, in northern Tanzania (Fig. 1). It is notable that the site, which preserves the most abundant assemblage of hominin footprints currently known from Africa, is within roughly 100 km of the site of Laetoli, which preserves the earliest confidently attributed hominin footprints^26^. The Engare Sero site was originally discovered by members of a Maasai community living nearby. In 2008, these individuals notified conservationists working in the area, and the conservationists brought the site to the attention of C.M.L.-P. When members of our research team first visited the site in 2009, an assemblage of 56 human footprints had already been exposed by natural surface erosion. From 2009 to 2012, our excavations (see Methods) exposed an additional 175 m^2^ of the footprint surface, which included at least 352 more human footprints (another 14 impressions were classified as “potential” human tracks). The same surface also preserves 19 bovid tracks in immediate association with the human footprints, and 24 zebra and buffalo tracks, which lie approximately 30 m to the southwest. Four additional assemblages of zebra tracks are located just over 1 km to the northwest, nearer the present-day shoreline of Lake Natron (Fig. 1).

The human footprint surface has been dated by this research team to between 5760 + /− 30 yrs BP and 19.1 + /− 3.1 ka, based on ^40^Ar/^39^Ar analysis as well as ^14^C radioisotopic dating techniques^24^. Another group suggests it may be closer to ~11,000 yrs BP^27^. Our team has built upon radioisotopic results to further constrain the likely age of the footprint surface to the latest Pleistocene, as we identified in previous analyses a calcite cement in an overlying sedimentary layer that was likely produced during a highstand of Lake Natron that occurred between 12 and 10 ka^24^. While an age at the very earliest Holocene is still possible, a latest Pleistocene age seems more probable based on the sum of available evidence from our analyses. Further details regarding the geological context and age of the site, as well as the depositional processes through which the footprint surface was formed and preserved, are published elsewhere^24,27^.

**Figure 2:**
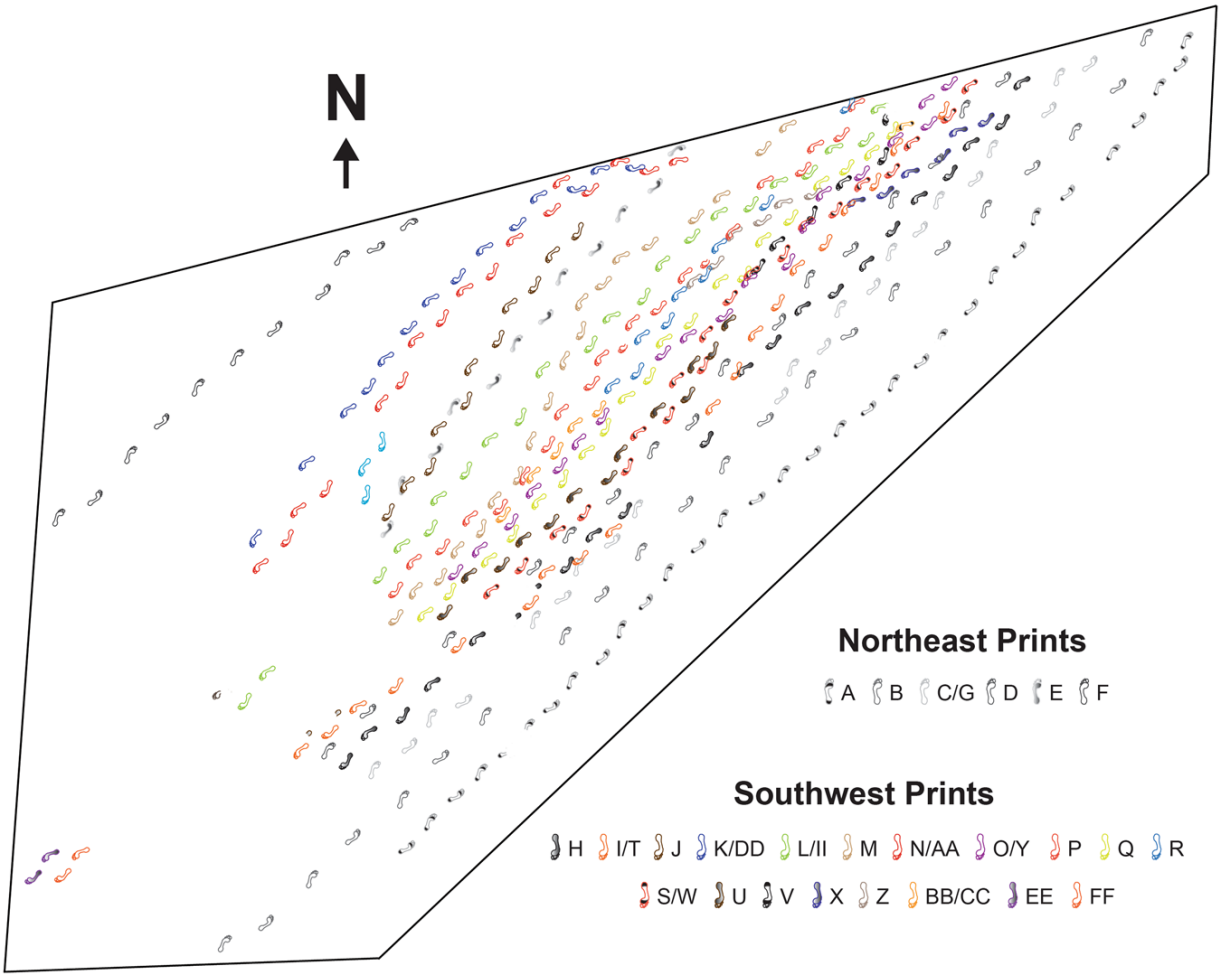
Schematic map showing the assemblage of human footprints preserved at Engare Sero. Footprints associated with the same trackway are indicated by a common color.

**Figure 3:**
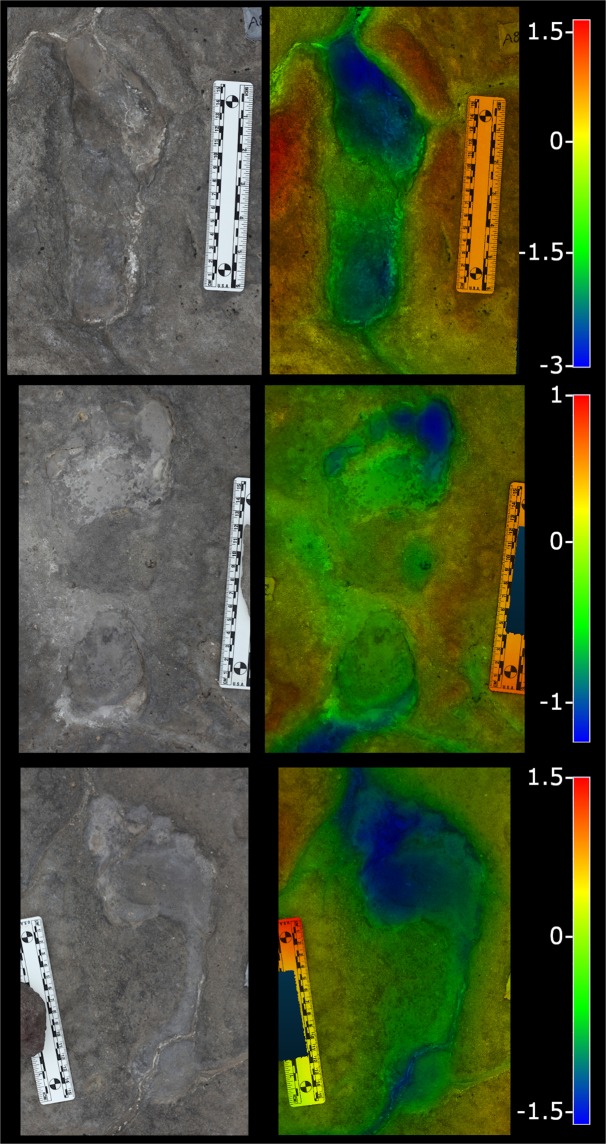
Examples of Engare Sero tracks. From top to bottom are tracks A8, I2, and D6. Overhead photographs are at left, orthographic images of 3D track models are at right, colored according to depth. Images are not set to common scale but each image includes a scale bar that is 15 cm in length. Color gradients that denote depth do not share a common scale and are instead specified to the right of each image set. The scales of those gradients are reported in centimeters.

As described above, the Engare Sero footprint surface preserves at least 408 human footprints (Fig. 2). All of the footprints appear to have been produced by barefoot humans, as individual toe impressions are easily distinguishable (Fig. 3). Because of their apparent human-like morphology and their Late Pleistocene age, we have attributed these tracks to *Homo sapiens*. There are likely many more than 408 tracks preserved at Engare Sero. Finite boundaries exist along three sides of the excavation, where surface sediments have been removed and no additional tracks are visible. However, along the northern boundary of the excavation, several trackways lead directly beneath unexcavated sediments (Fig. 2). Excavation of additional sediments has been halted until a long-term site conservation plan is implemented, as we know the exposed portion of the site is subject to erosion^25^.

## Results

### Estimating speeds of travel

**Figure 4:**
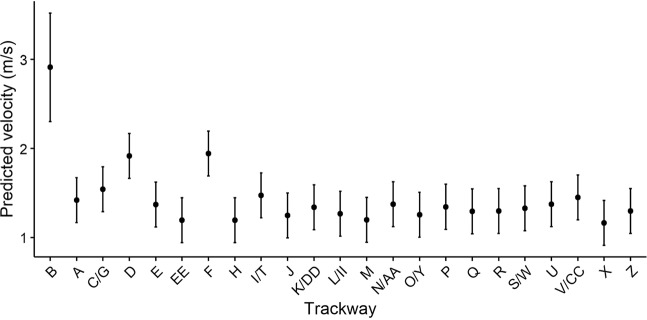
Speed estimates from Engare Sero trackways. Whiskers extending from the point estimates represent the root mean squared error of the relevant predictive models.

Speeds of travel were estimated for 23 distinct trackways from which we could obtain confident measures of stride length in addition to footprint length. In the first step of speed estimation (described in Methods), all but one of the 23 trackways appeared to reflect walking speeds (Table 1). This was not surprising, since relative stride lengths (stride length divided by foot length) of the trackways mostly fell between 5 and 6, while trackway B displayed a relative stride length greater than 8.5. Twenty of the trackways generated walking speed estimates that were quite similar, between about 1.2 to 1.5 m/s. Two trackways (D and F) appear to reflect slightly faster walking speeds (about 1.9 m/s), and we estimate that the individual who produced trackway B was running at 2.91 m/s. Notably, the 6 trackways oriented to the northeast show a broader range of variation in speed estimates, while the 17 trackways with southwestern orientations all appear to have been created at similar walking speeds between about 1.2 and 1.5 m/s (Fig. 4, Table 1).

**Table 1:** Estimated speeds of travel, compass orientations, and most probable age/sex group for each Engare Sero trackway^a^.

Trackway	Stride length/Footprint length	Predicted gait	Predicted velocity (m/s)	Compass direction	Compass orientation (degrees)	Age/sex attribution
A	5.69	Walk	1.42	NE	43	Adult female (70%)
B	8.58	Run	2.91	NE	46	Adult female (52%)
C/G	6.07	Walk	1.54	NE	46	Adult female (50%)
D	7.21	Walk	1.92	NE	49.5	Adult male (96%)
E	5.54	Walk	1.37	NE	43	Adult male (90%)
F	7.29	Walk	1.94	NE	49.5	Adult female (69%)
H	5.00	Walk	1.19	SW	235	Adult male (93%)
I/T	5.86	Walk	1.47	SW	226	Adult female (63%)
J	5.17	Walk	1.25	SW	218	Adult female (71%)
K/DD	5.45	Walk	1.34	SW	227	Adult female (61%)
L/II	5.23	Walk	1.27	SW	225	Adult female (57%)
M	5.01	Walk	1.20	SW	220	Adult female (69%)
N/AA	5.55	Walk	1.37	SW	228	Adult female (53%)
O/Y	5.19	Walk	1.26	SW	226	Adult male (83%)
P	5.46	Walk	1.34	SW	227	Adult female (52%)
Q	5.30	Walk	1.29	SW	225	Adult female (73%)
R/BB	5.32	Walk	1.30	SW	218.5	Adult female (70%)
S/W	5.41	Walk	1.33	SW	225	Adult female (65%)
U	5.56	Walk	1.38	SW	227	Adult female (55%)
V/CC	5.79	Walk	1.45	SW	226	Adult female (72%)
X	4.91	Walk	1.16	SW	233	Adult female (48%)
Z	5.31	Walk	1.30	SW	231	Juvenile male (45%)
EE	5.00	Walk	1.19	SW	—	Adult female (69%)

^a^Speed estimates are only provided for trackways in which reliable measurements of stride length could be obtained. Estimates of most probable age/sex group include in parentheses the percentage of resampled observations that corresponded to that particular attribution.

### Analyzing trackway orientations

An analysis of the compass orientations of trackways, using Rayleigh’s test of uniformity, revealed that both the northeast- and southwest-directed groups showed evidence of directionality (p < 0.05). The two groups traveled in almost perfectly opposite directions, with bootstrapped 95% confidence intervals ranging from 44 to 48.33 degrees for the northeast-directed group, and 223.94 to 228.41 degrees for the southwest-directed group. Trackway orientations are presented alongside walking speed estimates in Table 1.

### Estimating body size

**Figure 5:**
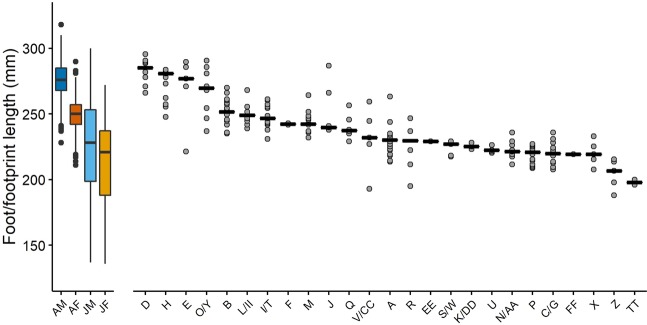
Median footprint lengths from Engare Sero trackways, alongside foot lengths from modern comparative data set. Solid line indicates the median length within each trackway. For comparative sample, the box includes the 25^th^ to 75^th^percentiles, the central bar indicates the median. The upper whiskers extend to 1.5 times the interquartile range, or the maximum value, whichever is smaller. The lower whiskers extend to 1.5 times the interquartile range, or the minimum value, whichever is larger. The following abbreviations are used for the comparative sample: AM = adult male (dark blue), AF = adult female (dark orange), JM = juvenile male (light blue), JF = juvenile female (light orange).

Summary statistics from the regression models that were ultimately used to predict stature are presented in Table S1. Stature estimates were possible for 25 trackways from which we could measure the relevant footprint dimensions. Median footprint lengths from these 25 trackways spanned the full range of foot sizes from the modern comparative sample (Fig. 5). Stature estimates ranged from heights that approached the tallest adult male individuals in the modern comparative sample (e.g., 1.83 m for trackway D) to heights that were comparable to small children in the modern comparative sample (e.g., 1.35 m for trackway TT). All stature estimates are presented in Table 2.

**Table 2:** Estimated statures for each Engare Sero trackway.

Trackway	Predicted stature (m)
A	1.53
B	1.66
C/G	1.47
D	1.83
E	1.78
EE	1.52
F	1.59
FF	1.47
H	1.80
I/T	1.62
J	1.58
K/DD	1.50
L/II	1.63
M	1.59
N/AA	1.48
O/Y	1.74
P	1.48
Q	1.57
R/BB	1.52
S/W	1.51
TT	1.35
U	1.48
V/CC	1.54
X	1.47
Z	1.40

### Estimating group structure

**Figure 6:**
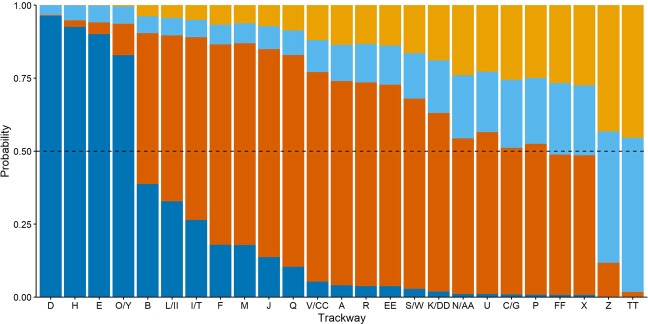
Probabilities that each Engare Sero trackway/track-maker could belong to each of the evaluated age/sex categories from the modern comparative data set. These categories include adult male (dark blue), adult female (dark orange), juvenile male (light blue), and juvenile female (light orange). Probabilities are calculated as the number of times a trackway was assigned to a given category in the resampling procedure, divided by 10,000 (the total number of iterations).

The results from a resampling protocol used to estimate group structure (see Methods) suggest that the Engare Sero trackways were created by a group that was mixed in terms of both age and sex (adult and juvenile females and males). Attributing each trackway to its most probable assignment would imply a group that included four adult males, 19 adult females, and two juvenile males (Fig. 6, Table S2). However, we emphasize that the two juvenile categories are less differentiable than the two adult categories (probabilities of juvenile male and juvenile female categorization are often very similar). There is also a significant degree of overlap in adult female and juvenile foot sizes. A footprint can therefore be attributed to the adult male category with greater confidence than to any of the other three groups used in this analysis. For example, trackways attributed to the adult male category are the only ones where classification probabilities exceed 90% (Table 1, Table S2).

The median resampled proportions for each age/sex category suggest a group structure that was roughly 20% adult males, 48% adult females, 16% juvenile males, and 16% juvenile females. These values are measures of central tendency, and do not come from the same iterative sample. The median adult sex ratio (number of males/number of females) was 0.45, suggesting slightly more than twice as many adult females than adult males within the group that created the Engare Sero trackways. The median adult to juvenile ratio was 2.57, suggesting roughly two and a half times as many adults as juveniles within the group. The median log adult footprint length dimorphism was 0.13. Resampled 95% confidence intervals for all of these calculations are provided in Table 3.

**Table 3:** Median estimates of age/sex proportions, adult sex ratio, adult to juvenile ratio, and log adult footprint length dimorphism, with resampled 95% confidence intervals.

Calculation	Median estimate	Lower 95% confidence limit	Upper 95% confidence limit
Adult male proportion	0.20	0.12	0.36
Adult female proportion	0.48	0.32	0.64
Juvenile male proportion	0.16	0.04	0.32
Juvenile female proportion	0.16	0.04	0.28
Adult sex ratio	0.44	0.20	1.00
Adult to juvenile ratio	2.13	1.08	7.33
Log adult footprint length dimorphism	0.13	0.09	0.17

## Discussion

Our predictions of walking speed suggest that 17 trackways with southwesterly orientations were created by individuals moving at walking speeds between 1.2–1.5 m/s (Fig. 4, Table 1). Such similarities in estimated walking speed suggest that these trackways could represent a group that was moving together. Other research has shown that humans tend to self-select optimal walking speeds that minimize energy expenditure^28^ but social and behavioral contexts may cause individuals to stray from their energetically optimal speeds. Notably, the speeds predicted from the Engare Sero trackways match well with experimental data on the speeds at which mixed-sex groups move when traveling together^29^. In their experiments, Wagnild and Wall-Scheffler^29^ found that male subjects self-selected walking paces greater than 1.5 m/s when walking alone or with other men but adjusted their paces to speeds below 1.5 m/s when walking with females. Since the collection of 17 Engare Sero trackways oriented towards the southwest were most likely created by a group that included both males and females (see below), this evidence lends support to the idea that the track-makers may have been walking together.

The six trackways with northeasterly orientations, however, generated a much wider variety of speed estimates. Two of track-makers were likely produced at faster walking speeds than are inferred from the southwesterly trails (about 1.9 m/s), while another likely reflects an individual who was running (a speed estimate of 2.9 m/s; Fig. 4, Table 1). Because of these differences in estimated speeds, it seems unlikely that the northeasterly trackways could represent a group that moved across the track surface together.

An analysis of the compass orientations of these trackways revealed that both the northeast- and southwest-directed groups showed evidence of common directionality (Rayleigh’s test of uniformity, p < 0.05). The two groups also traveled in almost perfectly opposite directions, with bootstrapped 95% confidence intervals ranging from 44 to 48.33 degrees for the northeast-directed group, and 223.63 to 227.44 degrees for the southwest-directed group. Despite their common orientations, trackways oriented in similar directions do not tend to overlap (i.e., they could have been made by individuals traveling abreast of each other). This adds further evidence to suggest that the group of trackways directed towards the southwest represents a collection of individuals who were traveling together. However, the fact that no trackways deviate substantially from either the northeast or southwest directions keeps us from definitively excluding the possibility that constraints on the natural landscape motivated common directions of travel.

Other analyses of hominin footprint sites in lake margin environments^20^ have found evidence that perhaps hominin foraging patterns involve transit that is parallel to lake shores. For example, Roach *et al*.^20^ found that hominin trackways at a *c*.1.5 Ma footprint site near Ileret, Kenya were parallel to each other and to the presumed lake shore, but approximately perpendicular to the trackways of bovids. Those authors inferred that hominins may have traveled along the shoreline to forage while bovids traveled to and from the water’s edge to drink, a pattern that they observed in modern humans and animals within present-day environments in the Turkana Basin^20^. At Engare Sero, unfortunately there are not many bovid trackways from which to assess the presence or absence of a similar pattern. The four bovid trackways that are immediately associated with the human tracks all seem to be oriented in the same southwest direction as the 17 human trackways (~210–230 degrees), a pattern that would contrast with that observed at Ileret. However, the zebra/buffalo tracks that appear roughly 30 m away from the rest of the assemblage are oriented in a direction perpendicular to the bovid and human trackways. Due to the small sample size of non-human tracks at Engare Sero, and difficulties in reconstructing the precise orientation of the southern shore of Lake Natron at the time when the tracks were produced, our abilities to draw behavioral inferences from these orientations are limited. For now, the most significant implication concerning behavior is that the large group of human trackways oriented toward the southwest seems to reflect individuals moving together in the same direction and at approximately the same speeds.

Estimates of stature that were derived from the Engare Sero trackways ranged from heights that correlate with tall adult males in the modern comparative sample (e.g., trackway D – 1.83 m) to those that correlate with children in the comparative sample (e.g., trackway TT – 1.35 m). These stature estimates match well with analyses of skeletal material from the latest Pleistocene and Holocene of East Africa, which have suggested generally tall and long-limbed body builds, similar to the statures observed in modern groups living in the same region^30^. However, skeletal material from this time period and region is generally scarce^31^, underscoring the value of the relatively large sample of anatomical data that is preserved on the Engare Sero footprint surface.

With respect to group composition, our analysis suggests that nearly half of the Engare Sero footprints were created by adult females (Table 3). Importantly, within the group of 17 southwest-directed trackways that we believe most likely reflects a group walking together, we calculate that 14 of those trackways were most likely produced by adult females (Table 1). Of the remaining three, we estimate that two were most likely produced by adult males and one by a juvenile male. This inferred group composition contrasts strongly with some that have recently been inferred from other fossil hominin footprint sites. For example, recent analyses of footprint sites attributed to *Homo heidelbergensis* and *H. neanderthalensis* have suggested that the evidence at each is consistent with mixed-age group structures and behaviors^21,23^. In addition to the apparent lack of children’s footprints, the continuous nature of the Engare Sero trackways also makes it unlikely that this snapshot of footprints similarly captures an occupation site^23^ or a location where food was being actively processed by both adult and juveniles^21^.

Earlier footprint assemblages attributed to *H. erectus* have been estimated to represent groups composed of 50% or more adult males who were possibly engaging in cooperative foraging behaviors^16,20,32^. While group composition is remarkably different among the Engare Sero assemblage, cooperative foraging remains a plausible hypothesis for the behavior that is represented on the footprint surface. Modern human foragers are unique among primates in that they typically forage together, and in that they typically divide labor between the sexes^33^. In modern human groups such as the Ache and Hadza, groups of adult females will cooperatively forage, with occasional visits or accompaniment from adult males^33,34^. Aside from nursing infants (who are likely to be carried), children are typically excluded from these types of group foraging activities and left behind in camp^33^. This scenario seems a plausible fit for the group structure and patterns of movements that are observed on the Engare Sero footprint surface. Other possibilities could certainly exist, however, and we look forward to the development of methods than can improve our abilities to accurately infer group behaviors from these forms of trace fossil data.

## Conclusions

The Engare Sero footprint assemblage provides a tantalizing snapshot of the movements of a group of modern humans living in East Africa in the Late Pleistocene. These trace fossils offer windows into anatomy, locomotion, and group behavior, which help to supplement what is known from other forms of fossil and archaeological data. They provide evidence of body sizes from a region and area where skeletal fossil data are scarce, and they preserve direct evidence of both walking and running behaviors. Within this assemblage is a collection of 17 trackways that is estimated to include at least 14 adult females traveling in the same direction and at similar speeds. This may represent direct fossil evidence of sexually divided foraging behaviors in Late Pleistocene humans. Such insights cannot be gleaned from most other forms of fossil data.

## Methods

### Excavation techniques, field measurements, and site documentation

Details on all field excavation and analysis methods are provided in the Supplementary Methods.

### Estimating speeds of travel

In our first step towards understanding the behaviors of the individuals whose footprints are recorded on the Engare Sero footprint surface, we wanted to estimate how fast various individuals were moving. For this analysis, we employed a data set collected from modern habitually barefoot people generating footprints at a variety of speeds. The experiments in which these data were collected have been described in detail elsewhere^35^. These experiments were carried out in accordance with the regulations of the George Washington University Institutional Review Board, and all participants provided their informed consent. Briefly, individuals walked across a ~ 15 m open-ended trackway, and produced footprints in a patch of hydrated mud at the center of that trackway. Three trials were conducted at each of four self-selected qualitative speeds – a normal walk, a fast walk, an endurance run, and a fast run. Video recordings of each trial were digitally analyzed to measure kinematic variables, including stride length and speed. The linear dimensions of the footprints that were generated in each trial were also measured. These same data have been used in the past to derive estimates of both speed and body mass from fossil hominin footprints^2,16^. Here, we derived velocity estimates using a procedure that partially differed from previous approaches, using a subset of the data that included measurements of velocity, stride length and footprint dimensions (n = 464 trials from 41 subjects). A detailed explanation of our approach is provided in the Supplementary Methods. These statistical analyses were conducted in R^36^, using custom scripts in addition to functions available in the dplyr^37^, caret^38^, nlme^39^, and ROCR^40^ packages (Supplementary Data S3).

### Analyzing trackway orientations

To assess the pattern in which the Engare Sero track-makers were moving, trackway orientations were also analyzed. Compass measurements from each trackway were analyzed using Rayleigh’s test of uniformity, to determine if they were oriented towards a common direction. One analysis was conducted on the entire data set, but since it was obvious that trackways were generally oriented in one of two directions (one directed to the northeast, the other to the southwest), separate analyses were also conducted on these two groups. Bootstrapped 95% confidence intervals of the average direction of travel, assuming a von Mises distribution, were also extracted. These statistical analyses were conducted in R^36^, using custom scripts in addition to functions available in the circular package^41^ (Supplementary Data S3).

### Estimating body size

Statures were estimated from the Engare Sero trackways, through statistical models that were built from the same experimental data set that was used to derive velocity predictions. In those experiments, the 41 subjects typically generated six footprints each in deformable mud, while traveling at walking speeds (n = 245; one observation was excluded due to a recording error during experimentation). Median footprint length (heel to hallux) and footprint breadths measured across both the forefoot and heel were extracted from each of the subjects’ prints, and used to derive regression equations for predicting stature. These methods are described in detail in the Supplementary Methods. These statistical analyses were conducted in R^36^, using custom scripts in addition to functions available in the dplyr^37^ and caret^38^ packages (Supplementary Data S3).

### Estimating group structure

To better understand the potential structure (i.e., composition) of the group of humans that created the Engare Sero footprints, we developed a statistical protocol to determine the likelihood that each trackway sampled an adult or juvenile female or male individual. This analysis required two different comparative datasets. The first of these was the same experimental footprint data set described above, which was used for speed and stature estimation. The second included data from a large sample of 3233 individuals (1652 male, 1581 female, ranging from 2 to 52 years of age), which were collected during two published anthropometric studies^42,43^ and made available to us. This data set included measurements of foot length for each participant in the study and identified whether each subject was male or female. We note that this anthropometric study did not describe the methods that were used for sex categorization. Sex classifications restricted to only two categories do not appropriately consider biological and anatomical diversity related to sex, and we acknowledge that by employing this comparative data set these limitations will carry over to our fossil analysis.

In the first step of this analysis, the experimental footprint data set was used to better understand variation in footprint size relative to foot size. When considering proportional differences between footprint length and foot length (relative to foot length), the experimental footprints fell within about 10% less than foot length to 15% more than foot length (Fig. S1). We compared this range to the proportional differences between individual Engare Sero footprints and the median footprint length of the trackway to which they belong (Fig. S2). We found that the range of variation around the median of each trackway is about the same or less than the range of variation of experimental prints around true foot length. (However, among the fossil tracks we tended to see more footprints much smaller than the trackway median rather than much larger). The median footprint length is therefore likely to be roughly equivalent to foot length if we include a proportional constant (which is likely dependent upon the substrate in which a footprint is formed) and an error term. Unraveling this equation was not the objective of the current study, so we proceeded with the assumption that an individual’s foot length is likely to fall within roughly 5% (smaller or larger) of that same individual’s median footprint length. This assumption was supported by our experimental data, in which footprints were on average about 2% larger than true foot size (Fig. S1). Very few footprints (less than 5%) were more than 5% shorter than true foot length. Roughly 23.5% of footprints were more than 5% longer than true foot length, but this tended to occur at higher speeds of travel. For speeds below 1.47 m/s (the highest estimate obtained from the southwest-oriented Engare Sero group that seems to be traveling together), there were only seven cases in which a footprint was more than 5% longer than true foot size.

Next, we sought to develop a method for distinguishing adult versus juvenile feet, this time employing the much larger ontogenetic anthropometric data set. First, we developed sex-specific growth plots for foot length, and visually identified cessations of foot growth at approximately age 14 for females and age 17.8 for males (Fig. S3). All individuals equal in age or older than those sex-specific cutoffs in this sample are considered to have adult feet, and all younger individuals have juvenile feet. These classifications were then used in the process of classifying footprints to age-sex categories.

Our classification procedure for the Engare Sero trackways began by calculating the median footprint lengths for each individual trackway. We assumed that the individual that made the trackway with the largest median footprint length (trackway D, median length = 289.5 mm) had a foot that was at least one standard deviation longer than the adult male mean foot length for the population to which that individual belonged. In order to match foot lengths from the modern comparative sample to the fossil prints, we calculated in the comparative sample the adult male foot length mean and standard deviation. In the iterative procedure, we used the comparative mean plus one standard deviation as a lower cutoff for foot lengths from which we will sample a length to represent the largest fossil individual in each iteration. For all other trackways, we calculated the median footprint length as a proportion of the median length from the largest Engare Sero track-makers. We then conducted an iterative resampling procedure as follows.

We noted that the age distribution for the comparative sample is heavily biased towards 18- to 34-year-olds, with a particularly high proportion of 18- to 20-year-olds (Fig. S4). The first step of the iterative procedure was to separate the comparative sample into two-year age intervals for each sex, and any sex-specific interval which had more than 50 individuals was randomly sampled down to 50 individuals without replacement. This generated a more uniform age distribution, and effectively increased the probability that juvenile feet would be sampled if they fell within the correct size window (Fig. S5).

A different random sample was generated in each of 10,000 iterations of the procedure described below. In the first step, we chose at random a foot from the reduced comparative sample that was at least as long as the lower cutoff value for the largest individual in the sample for this particular iteration (as described above). For each additional trackway, we multiplied the proportional lengths of the upper and lower bounds on print size for that trackway (lower bound = 95% of proportional median footprint length, upper bound = 105% of proportional median footprint length) by the length of the randomly selected foot representing the largest trackway. For each trackway, these minimum and maximum values bracket a set of feet in the comparative sample from which we would randomly select one foot to represent that trackway. For each selected foot in the random sample for a particular iteration, we noted the sex, age, and length of the foot. For the random sample, we then calculated the proportion of adult males, adult females, juvenile males, and juvenile females that were sampled. We also calculated the log sex ratio (log of the number of males divided by number of females) and the log dimorphism ratio (log of mean male foot length divided by mean female foot length) in each iteration. This suite of steps was then repeated 10,000 times.

We calculated 95% confidence intervals for the proportion of adult males, adult females, juvenile males, and juvenile females represented in the Engare Sero trackways” by sorting the 10,000 proportions for each age-sex category and discarding the highest and lowest 2.5%. We calculated 95% confidence intervals for the adult sex ratio, adult to juvenile ratio, and log adult footprint length dimorphism (log[mean adult male foot size/mean adult female foot size]) in the same way.

These statistical analyses were conducted using custom scripts in R^36^ that also made use of functions from the Hmisc^44^ and reshape2^45^ packages (Supplementary Data S3).

## Supplementary information

Supplementary Data S1

Supplementary Data S2

Supplementary Data S3

Supplementary information.

Supplementary information5.

## Data Availability

The authors declare that all original data and code for analyses that support the findings of this study are available within the paper and its supplementary files. Certain experimental data were obtained from previously published studies. Because they are not original to this study, those raw data are not included here, however the references to the original studies are provided.
