# Supplementary material for: Snapshots of human anatomy, locomotion, and behavior from Late Pleistocene footprints at Engare Sero, Tanzania
Source: Sci Rep. 2020 May 14;10:7740. doi: 10.1038/s41598-020-64095-0 (PMC7224389; doi:10.1038/s41598-020-64095-0)
Supplement: Supplementary file 4 — Supplementary information. [file 41598_2020_64095_MOESM4_ESM.docx]

**Description of supplementary files**

File name: Supplementary Information

Description: Supplementary text, figures, and tables to accompany the main text of the article.

File name: Supplementary Data S1

Description: Table with measurements of all Engare Sero tracks. Measurements include linear distances from heel to the tip of digit 3, heel to the tip of the hallux, forefoot breadth, and heel breadth, all measured in millimeters. Measurements follow the template published by Bennett and Morse^1^. Not all measurements were possible for all tracks, and some tracks precluded reliable measurement. Only measurements made with confidence are included in this data table.

File name: Supplementary Data S2

Description: Table with compass orientations and median stride lengths for all Engare Sero trackways. Median stride lengths are measured in centimeters. One trackway (EE) lacks a compass orientation measurement.

File name: Supplementary Data S3

Description: R code that was used to conduct analyses and generate figures.
