# Supplementary material for: Snapshots of human anatomy, locomotion, and behavior from Late Pleistocene footprints at Engare Sero, Tanzania
Source: Sci Rep. 2020 May 14;10:7740. doi: 10.1038/s41598-020-64095-0 (PMC7224389; doi:10.1038/s41598-020-64095-0)
Supplement: Supplementary file 5 — Supplementary information5. [file 41598_2020_64095_MOESM5_ESM.pdf]

Supplementary Information

Snapshots of human anatomy, locomotion, and behavior from Late Pleistocene footprints at Engare Sero, Tanzania

Kevin G. Hatala, William E.H. Harcourt-Smith, Adam D. Gordon, Brian W. Zimmer, Brian G. Richmond, Briana L. Pobiner, David J. Green, Adam Metallo, Vince Rossi, Cynthia M. Liutkus-Pierce

Supplementary Methods

Excavation techniques

As described in the main text, at the time of discovery the site had already been partially exposed by natural erosional processes. The 2009 field season focused on initial documentation of the exposed surface and its context, and our research team began excavating the site in 2010. During the 2010 field season, we swept away loose sediment that was overlying the footprint surface, using soft bristled brooms. No lithified sediments were removed in these initial excavations. Using this technique, we uncovered approximately 300 additional human footprints. In 2012, we returned to the site to continue our excavations, measurements, and analyses. Our excavations in this year included removal of additional loose sediment but also small blocks of the lithified mica-rich layer overlying the footprint surface (MRL). These blocks of the MRL were immediately overlying the footprint surface but in the midst of eroding away along the northern border of our excavation. The additional cleaning and exposure of the surface in 2012, combined with a re-examination of previously excavated areas, further increased the sample of exposed hominin tracks by approximately 50. From 2013 to 2017, geological work and

laboratory analyses continued in order to determine the age and context of the site[1], however, no excavations were conducted after 2012.

Field measurements of tracks and trackways

Using portable equipment, immediately after excavation we collected a variety of field measurements from each track and trackway (a series of sequential tracks made by the same individual). The length of each track was measured with a tape measure in two ways – from the most proximal point of the heel impression to the most distal point of the hallux impression, and from the most proximal point of the heel impression to the most distal point of the third toe impression. Widths of tracks were also measured using a tape measure both across the forefoot (between the lateral and medial walls of the track adjacent to the estimated locations of the first and fifth metatarsal head impressions) and the heel. The compass orientation of each individual track was measured using a Brunton international pocket transit (Brunton, Louisville, CO, USA).

Once all tracks were excavated, we evaluated the sizes, shapes, positions, and orientations of the tracks in order to link sequential tracks together and assign them to specific trackways (Fig. 2). After assigning tracks to trackways, stride- and step-lengths were measured using metric tape measures. These lengths were typically measured from heel-to-heel of sequential tracks, although in some cases measurements from toe-to-toe were necessary due to poor definition of heel impressions. The compass orientations of each trackway were also measured directly. A string was positioned and held taught such that it ran in between the tracks associated with a given trackway. The compass orientation of that string was then measured using a Brunton pocket transit. All track and trackway measurements are provided in Supplementary Data 1 and 2, respectively.

Digital 3D documentation of site

During the 2010 field season, the entire exposed footprint surface was documented in 3D using photogrammetry, in accordance with standard protocols developed for the study of fossil tracks[2]. A Canon 5D Mark II DSLR camera, fitted with a Canon EF 50 mm prime lens, was mounted to an adjustable tripod with a boom arm, such that overhead photos could be taken from a height of approximately 2 meters. The tripod was moved across the footprint surface and sequential overhead photographs were taken, with approximately 2/3 overlap between adjacent images. The camera was remotely controlled from a laptop, such that images could be previewed before capture. During photographic capture, the portion of the surface that was in view of the camera was shaded with a portable tent, in order to provide consistent lighting. Tape measures and scale bars were placed across the surface prior to photography, in order to ultimately scale the 3-D model that would be generated from the photographs. Certain high-fidelity footprints were also photographed by hand from even more heights and different angles, to provide even higher resolution of those areas. Following the 2010 field season, images of the footprint surface were processed using Adam Technology 3DM Analyst software (www.adamtech.com.au), which stitched together all photos in order to generate a 3D photogrammetric model of the entire exposed footprint surface, with sub-millimeter accuracy.

In 2012, a similar camera (Canon 5D Mark II, Canon EF 50 mm prime lens) was used to photograph by hand all newly exposed human and other animal footprints. Approximately 15-25 scaled photographs were taken of each sufficiently preserved human footprint, from a variety of heights and angles (the number of photographs increased with the depth or topographic complexity of the tracks, in an attempt to ensure consistent model accuracy). In 2017, a Canon Powershot SX60 HS camera with adjustable lens was used to photograph and model a selection

of footprints, in order to monitor erosion of the footprint surface that may have occurred since its initial excavation[3].

Estimating speeds of travel

In order to derive predictions of velocity from the information recorded within the trackways, we first needed to derive an appropriate statistical model. Since taller individuals take longer strides than shorter individuals, we standardized all experimental and fossil trackway stride length measurements by the lengths of the tracks that comprise a given trackway. For our experimental data set, this meant that each stride length measurement was divided by the length of the footprint that was directly produced by that stride (i.e., relative stride lengths were calculated per trial). For the Engare Sero footprints, this method was less appropriate because each trackway included certain tracks and/or stride lengths that could not be measured due to differential preservation (e.g., a particular track was obliterated by an overlapping track from another individual, or a trackway might proceed from a right track to another right track with the intervening left track not preserved at all). Instead, we calculated the median stride length and the median track length within each trackway. Track lengths and median stride lengths are presented in Supplementary Data 1 and 2, respectively.

Using the experimental data, we constructed a simple linear regression model of velocity by relative stride length that included all walking and running trials. The model was built on a training data set that included a randomly selected 70% of the experimental observations, and the predictive accuracy of the model was then tested by deriving predictions for the remaining 30% of trials. The root mean square error (RMSE) of velocity predictions derived from that model was found to be 0.45 m/s.

A second approach was then tested, in which we assessed whether more accurate velocity predictions could be obtained by first predicting gait type (walking or running) and then deriving numerical speed predictions from a "walk-only" or "run-only" regression of velocity by relative stride length. The data were divided as before, with a randomly-selected 70% of trials designated as the training set and the other 30% as the test set. Using the training data, a logistic regression model was used to model the binary outcome of gait type (walk or run) as a function of relative stride length. Two separate linear regression models of velocity by relative stride length were then constructed using data from walking and running trials. Within the test data set, the logistic regression model showed a gait classification accuracy rate of 97.79%. By using this model to first predict gait type and then analyzing those test data through the relevant linear regression models ("walk-only" or "run-only", using the predicted gait types), RMSE for walking velocities was reduced to 0.25 m/s while that of running velocities increased to 0.61 m/s. In the latter case, the greater RMSE for predicting running velocities may be linked to greater between-subject variation in running versus walking mechanics. For example, habitually barefoot people are known to use a variety of foot strike patterns during running[4–6]. Those runners who use different foot strike patterns typically differ in other aspects of lower limb kinematics including stride frequency, which is inversely related to stride length for a given speed[7]. Therefore, one might expect greater error when predicting velocity from stride length during running bouts. Since this approach resulted in more accurate walking velocity predictions, and all but one of the fossil trackways displayed relative stride lengths that were consistent with walking speeds (see Results), we preferred this regression model for predicting speeds of travel.

Finally, because the experimental data were collected using a repeated measures study design (the same subjects each walked and ran multiple times), we also tested whether a mixed

effects model might generate more accurate predictions. Using the same training and test data sets described in the preceding paragraph, we constructed and then evaluated predictions from mixed effects models that included subject identity as a random effect on the intercept and/or slope. Using model selection and validation methods described by Zuur et al.[8], we found that modeling subject identity as a random effect on the intercept and slope of the model led to a better model fit (evaluated via Akaike's Information Criterion) with the training data. Predictions were then generated for the test data using population parameters from the mixed effects model. These predictions resulted in RMSE values of 0.24 m/s and 0.60 m/s for the "walk-only" and "run-only" regression models, respectively, slight improvements over the models that did not account for random effects. Population parameters from the mixed effects model were used to generate velocity predictions for the 23 Engare Sero trackways from which relative stride lengths could be measured. These statistical analyses were conducted in R[9], using custom scripts in addition to functions available in the dplyr[10], caret[11], nlme[12], and ROCR[13] packages (Supplementary Data S3).

Estimating body size

To derive estimates of body size, experimental data on footprint sizes and statures were divided into training and test sets to evaluate error rates of predictions (70% of data were randomly assigned to the training set, while the other 30% were assigned to the test set). A multiple regression model was fit to the data, with stature as the dependent variable and three footprint dimensions (length from heel to hallux, forefoot breadth, and heel breadth) as independent variables. Following normal model selection protocols (removing non-significant predictors and iteratively re-evaluating model fit), the best overall model fit was provided by a

model that included both footprint length and forefoot breadth as independent variables. However, evaluations with the test data revealed that a model that used footprint length alone to predict stature actually generated the most accurate predictions (RMSE of 6.27 cm, compared with 7.66 cm or 7.68 cm respectively in models that also included forefoot breadth, or forefoot breadth and heel breadth). Since predictive accuracy was considered the most important evaluative criteria, we proceeded to predict statures from the Engare Sero trackways using the model that only included footprint length.

The same procedure was repeated to predict stature from footprints created during bouts of running, as different gaits might influence how particular footprint dimensions relate to stature. Each of the same 41 subjects also typically generated six footprints each at running speeds (n = 245; one observation was again excluded due to a recording error during experimentation). The median running footprint dimensions were extracted for each subject, and a multiple regression model was again fit to predict stature from the same footprint dimensions. Again, a randomly chosen subset (70%) was used to train the model, while the remaining data (30%) were held out for testing predictive accuracy. The model including both footprint length and forefoot breadth once again generated the best overall fit, evaluated via AIC. However, the model including footprint length as the sole independent variable generated yet again the most accurate predictions (RMSE of 7.82 cm, compared with 8.23 cm or 8.47 cm respectively in models that also included forefoot breadth, or forefoot breadth and heel breadth). Therefore, for both walking and running trackways from Engare Sero, only the median footprint lengths were used to predict stature.

Supplementary Figures

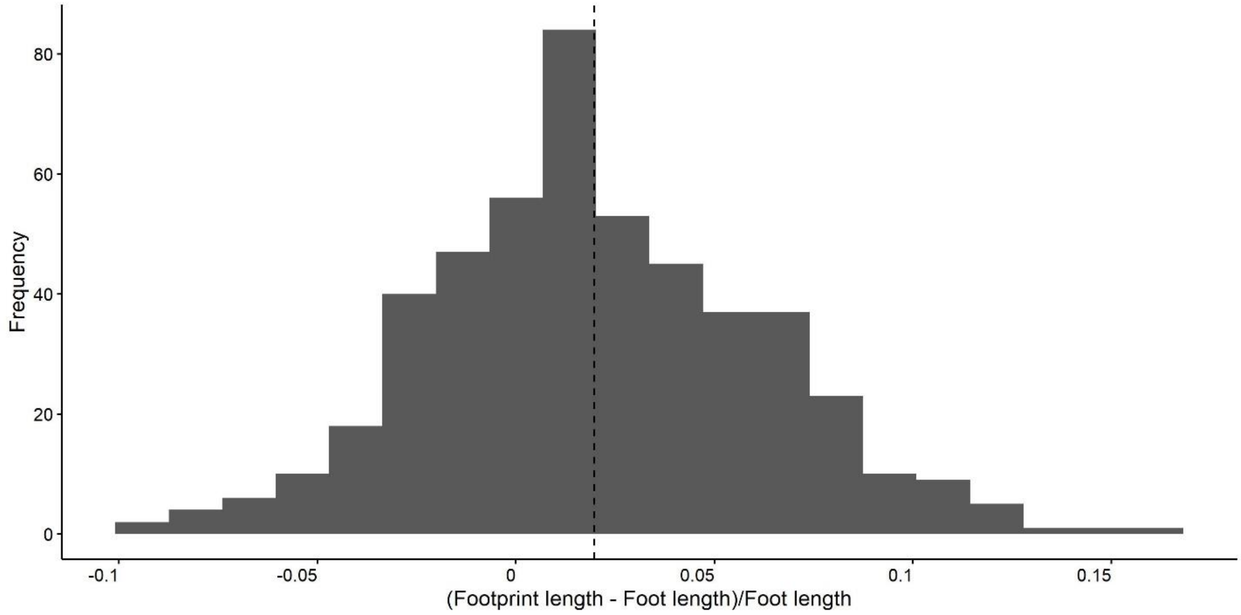

Figure S1. Histogram displaying proportional differences between footprint and foot lengths (both measured from heel to hallux) from a modern human experimental sample. Footprints are more frequently longer than true foot length rather than shorter. The dashed line indicates the mean proportional difference of approximately 0.02.

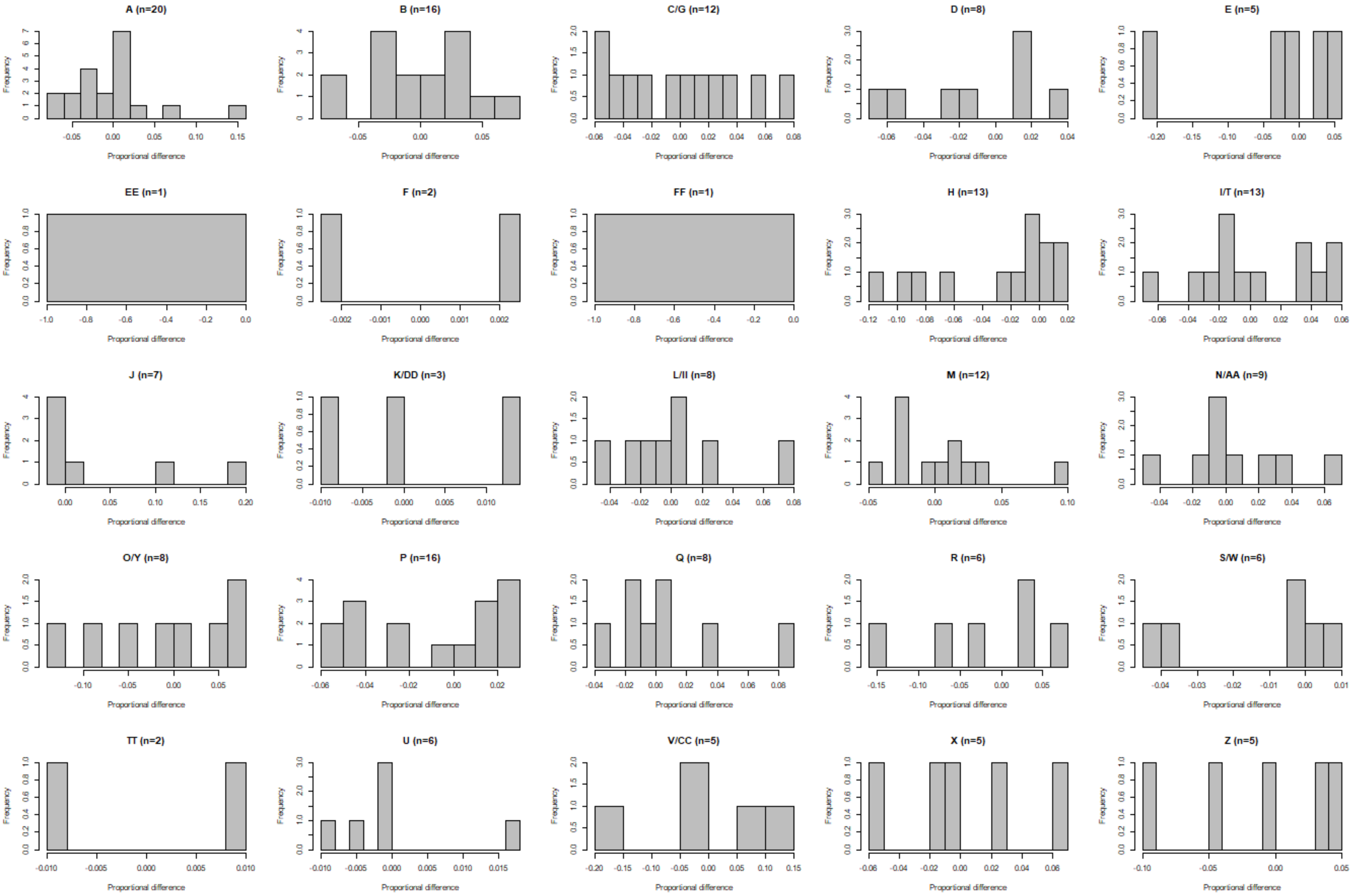

Figure S2. Histograms displaying proportional differences between individual footprint lengths and the median lengths from within their respective trackways. The number of tracks whose lengths could be confidently measured from each trackway is provided in parentheses.

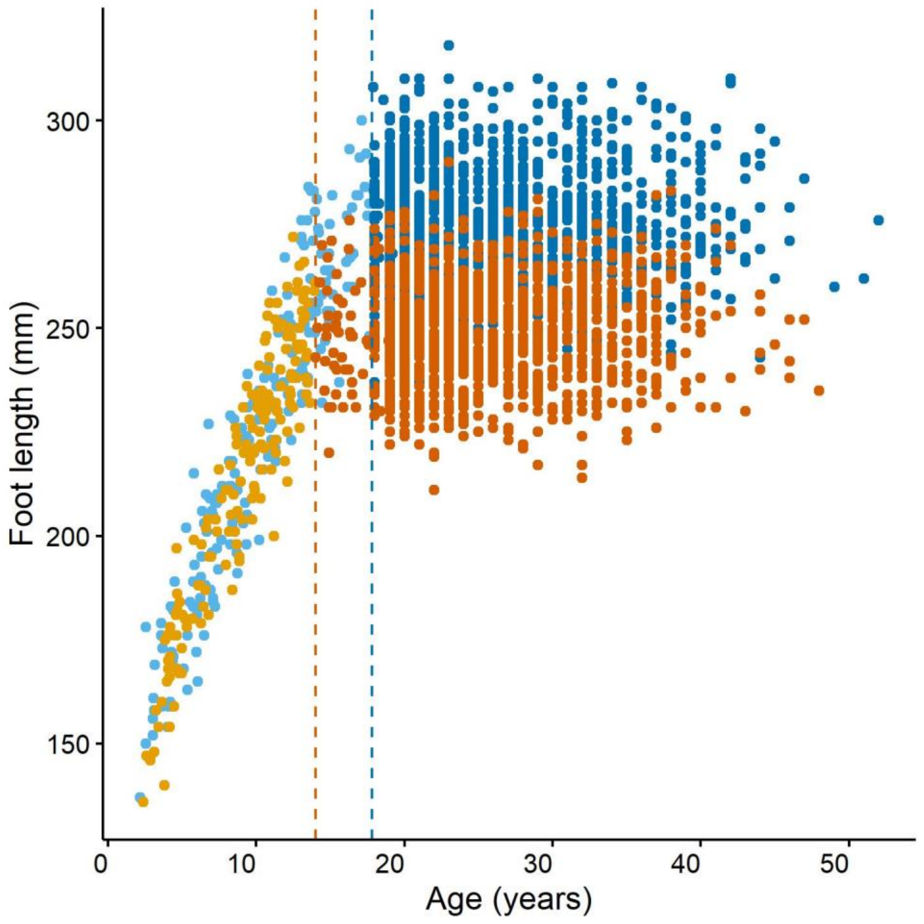

Figure S3. Scatterplot showing cross-sectional ontogenetic sample of modern human foot lengths from previously published anthropometric studies[14,15]. Adult males are plotted in dark blue, adult females in dark orange, juvenile males in light blue, and juvenile females in light orange. Based on visual inspection of these data, cessations of growth in foot length were identified at roughly 14 years for females and 17.8 years for males. These ages are indicated by the dark orange and dark blue dashed lines, respectively.

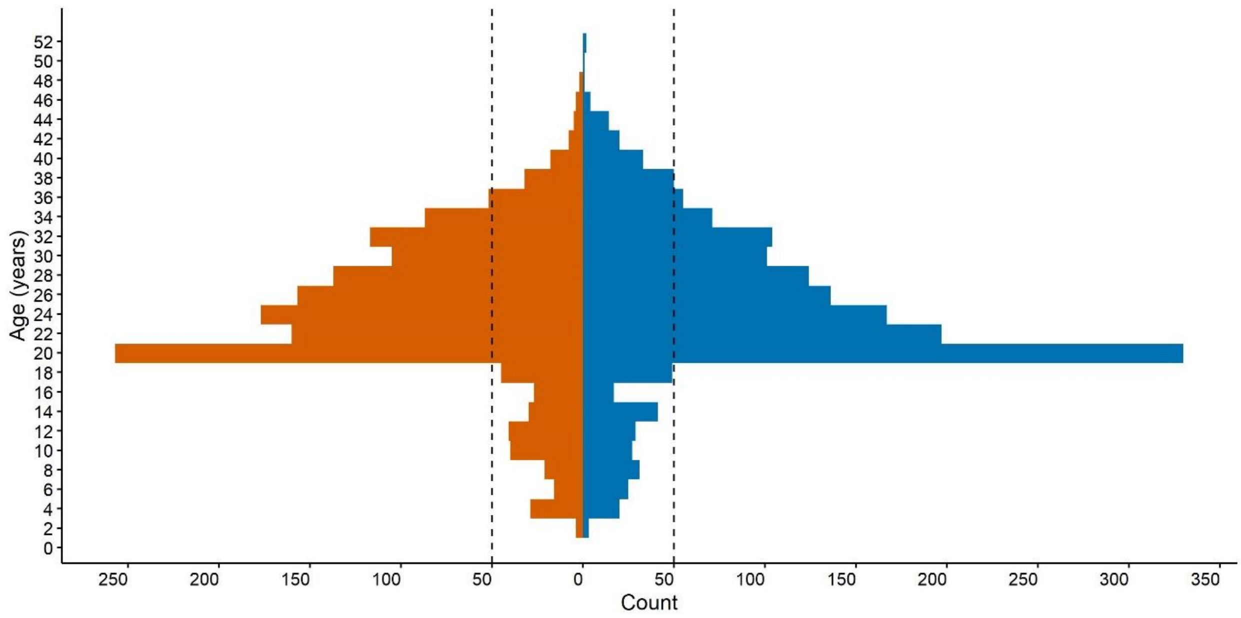

Figure S4. Histograms showing the age distribution of the total sample of foot lengths obtained from previously published anthropometric studies[14,15]. Females are plotted in orange and males are plotted in blue. The data set is clearly biased towards individuals between roughly 18 and 34 years of age, with a particularly large cluster of 18- to 20-year-olds. Dashed lines indicate the cut-off for producing a more balanced comparative sample for each iteration, such that the comparative samples include no more than 50 individuals from any two-year age interval.

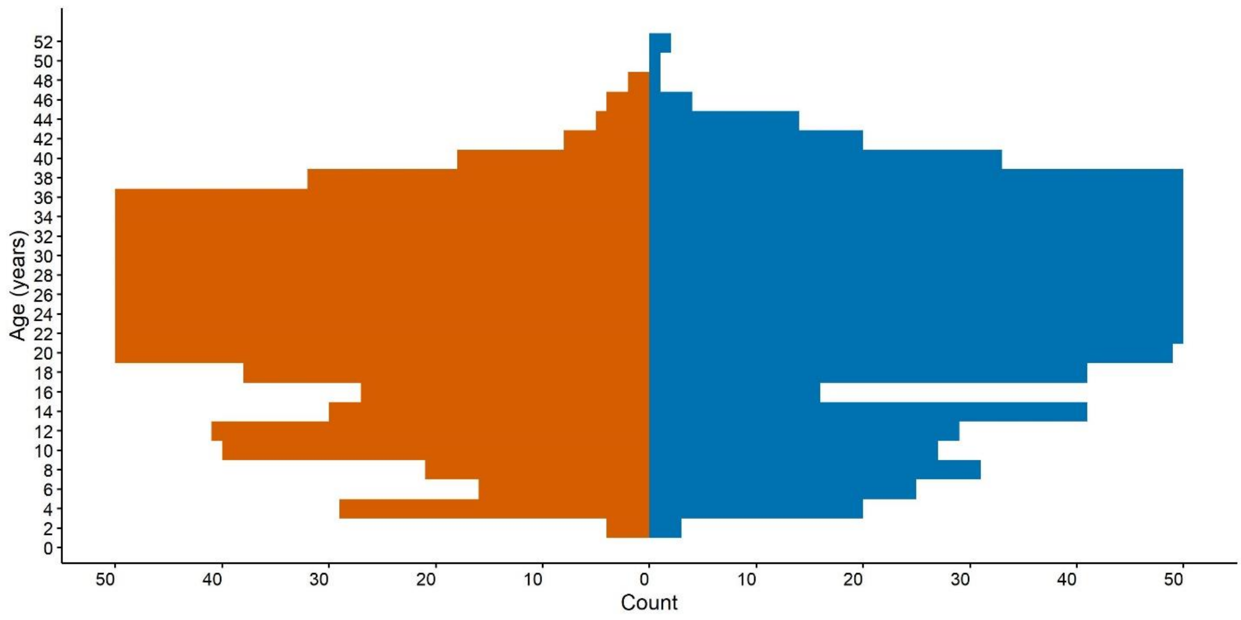

Figure S5. Histograms showing the age distribution of the reduced sample of foot lengths obtained from previously published anthropometric studies[14,15]. The sample was reduced in order to achieve a relatively more balanced age distribution, with no more than 50 individuals falling in any two-year age intervals. Females are plotted in orange and males are plotted in blue.

Supplementary Tables

Table S1. Summary statistics from regression models used to predict stature from walking and running footprint dimensions.

Variable	Effect size	Standard error	P-value
	Walking model (adjusted R^2 = 0.62)		
Intercept	28.56	19.58	0.16
Footprint length	5.33	0.76	$1.39*10^{-7}$
	Running model (adjusted R^2 = 0.80)		
Intercept	22.81	13.19	0.09
Footprint length	5.61	0.52	$1.69*10^{-11}$

Table S2. Probabilities of age/sex categories for each Engare Sero trackway.[a]

Trackway	Adult male	Adult female	Juvenile male	Juvenile female
A	4.07	**69.93**	12.37	13.63
B	38.70	**51.67**	5.81	3.82
C/G	0.94	**50.17**	23.35	25.54
D	**96.36**	0.25	3.39	0.00
E	**90.07**	4.03	5.75	0.15
EE	3.74	**68.99**	13.44	13.83
F	17.89	**68.67**	6.74	6.70
FF	0.72	**48.08**	24.48	26.72
H	**92.56**	2.23	5.13	0.08
I/T	26.36	**62.63**	5.95	5.06
J	13.69	**71.25**	7.86	7.20
K/DD	1.88	**61.18**	18.05	18.89
L/II	32.79	**56.80**	6.04	4.37
M	17.83	**69.07**	6.87	6.23
N/AA	1.07	**53.30**	21.65	23.98
O/Y	**82.85**	10.81	5.90	0.44

P	0.85	**51.68**	22.53	24.94
Q	10.36	**72.57**	8.37	8.70
R/BB	3.76	**69.76**	13.20	13.28
S/W	2.85	**65.14**	15.57	16.44
TT	0.00	1.73	**52.88**	45.39
U	1.05	**55.46**	20.72	22.77
V/CC	5.29	**71.82**	10.89	12.00
X	0.71	**47.88**	23.97	27.44
Z	0.04	11.75	**44.93**	43.28

[a]Probabilities are calculated as the percentages of classifications into the given category through the resampling protocol. The most probable classification for each trackway is displayed in bold text. Two trackways shown here (FF and TT) do not appear in Table 1 of the main text, because measurements of stride length and trackway orientation were not possible.

References

1. Liutkus-Pierce, C. M. *et al.* Radioisotopic age, formation, and preservation of Late Pleistocene human footprints at Engare Sero, Tanzania. *Palaeogeogr. Palaeoclimatol. Palaeoecol.* **463**, 68–82 (2016).

2. Falkingham, P. L. *et al.* A standard protocol for documenting modern and fossil ichnological data. *Palaeontology* **61**, 469–480; https://doi.org/10.1111/pala.12373 (2018).

3. Zimmer, B. *et al.* Using differential structure-from-motion photogrammetry to quantify erosion at the Engare Sero footprint site, Tanzania. *Quat. Sci. Rev.* **198**, 226–241 (2018).

4. Lieberman, D. E. *et al.* Foot strike patterns and collision forces in habitually barefoot versus shod runners. *Nature* **463**, 531–535 (2010).

5. Hatala, K. G., Dingwall, H. L., Wunderlich, R. E. & Richmond, B. G. Variation in foot strike patterns during running among habitually barefoot populations. *PLoS One* **8**, e52548; https://doi.org/10.1371/journal.pone.0052548 (2013).

6. Pontzer, H. *et al.* Foot strike patterns and hind limb joint angles during running in Hadza hunter-gatherers. *J. Sport Heal. Sci.* **3**, 95–101 (2014).

7. Lieberman, D. E. *et al.* Variation in foot strike patterns among habitually barefoot and shod runners in Kenya. *PLoS One* **10**, e0131354; https://doi.org/10.1371/journal.pone.0131354 (2015).

8. Zuur, A. F., Ieno, E. N., Walker, N., Saveliev, A. A. & Smith, G. M. *Mixed effects models and extensions in ecology with R*. (Springer, 2009).

9. R Core Team. *R: A language and environment for statistical computing, Version 3.6.1*.

http://www.R-project.org (R Foundation for Statistical Computing, 2019).

10. Wickham, H., François, R., Henry, L. & Müller, K. *dplyr: a grammar of data manipulation, Version 0.8.3*. http://dplyr.tidyverse.org/ (CRAN, 2019).

11. Kuhn, M. *et al. caret: classification and regression training, Version 6.0-84*. https://github.com/topepo/caret/ (CRAN, 2019).

12. Pinheiro, J., Bates, D., DebRoy, S., Sarkar, C. & R Core Team. *nlme: linear and nonlinear mixed effects models, Version 3.1-141*. https://svn.r-project.org/R-packages/trunk/nlme (CRAN, 2019).

13. Sing, T., Sander, O., Beerenwinkel, N. & Lengauer, T. ROCR: visualizing classifier performance in R. *Bioinformatics* **21**, 7881 (2005).

14. Gordon, C. C. *et al.* 1988 Anthropometric survey of U.S. army personnel: Methods and summary statistics. (US Army Natick Research, Development and Engineering Center, 1989).

15. Snyder, R. G. *et al.* Anthropometry of infants, children, and youths to age 18 for product safety design. (Consumer Product Safety Commission, 1977).
